# Wavelet-CNet: Wavelet Cross Fusion and Detail Enhancement Network for RGB-Thermal Semantic Segmentation

**DOI:** 10.3390/s26031067

**Published:** 2026-02-06

**Authors:** Wentao Zhang, Qi Zhang, Yue Yan

**Affiliations:** 1 School of Mathematics and Computer Science, Shaanxi University of Technology, Hanzhong 723001, China; zhangwentao@snut.edu.cn (W.Z.);; 2 College of Computer Science, Northwest University, Xi’an 710127, China

**Keywords:** RGB-T semantic segmentation, wavelet transform, cross scale, bilateral network

## Abstract

Leveraging thermal infrared imagery to complement RGB spatial information is a key technology in industrial sensing. This technology enables mobile devices to perform scene understanding through RGB-T semantic segmentation. However, existing networks conduct only limited information interaction between modalities and lack specific designs to exploit the thermal aggregation entropy of the thermal modality, resulting in inefficient feature complementarity within bilateral structures. To address these challenges, we propose Wavelet-CNet for RGB-T semantic segmentation. Specifically, we design a Wavelet Cross Fusion Module (WCFM) that applies wavelet transforms to separately extract four types of low- and high-frequency information from RGB and thermal features, which are then fed back into attention mechanisms for dual-modal feature reconstruction. Furthermore, a Cross-Scale Detail Enhancement Module (CSDEM) introduces cross-scale contextual information from the TIR branch into each fusion stage, aligning global localization through contour information from thermal features. Wavelet-CNet achieves competitive mIoU scores of 58.3% and 85.77% on MFNet and PST900, respectively, while ablation studies on MFNet further validate the effectiveness of the proposed WCFM and CSDEM modules.

## 1. Introduction

**Figure 1:**
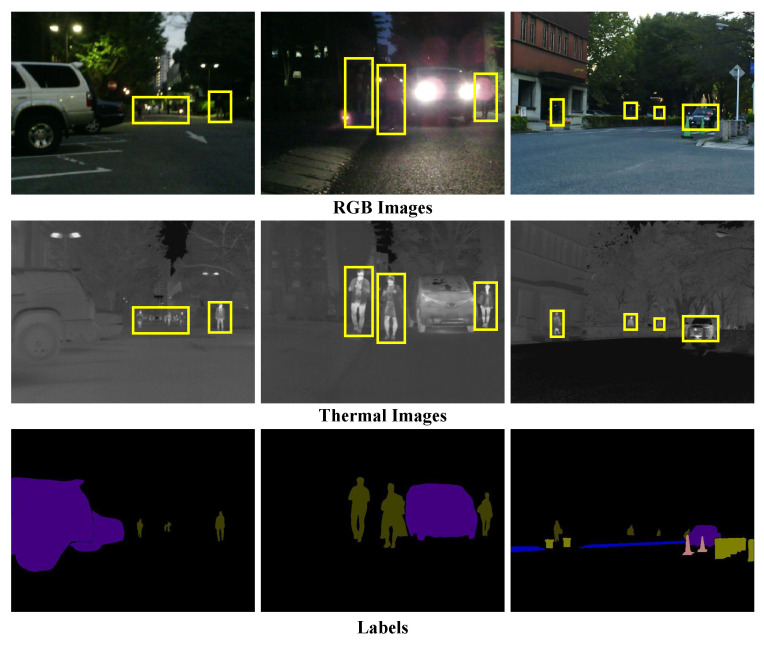
Information discrepancy between RGB and thermal images. As highlighted by the yellow boxes, certain target objects suffer from degraded visual perception due to lighting variations, shadow interference, and occlusions. In contrast, thermal images clearly delineate the contours and positions of heat-emitting subjects.

Computer vision and robotic systems typically rely on visible-light sensors to capture color information from real-world scenes for semantic understanding and scene analysis [1,2,3,4,5,6,7,8]. However, unimodal RGB imaging suffers from inherent limitations in complex environments, as its image quality is highly dependent on illumination conditions and sensor performance and is easily degraded by occlusion, adverse weather (e.g., haze), and motion blur, leading to structural detail loss and reduced robustness of deep learning models. In contrast, thermal imaging captures scenes based on infrared radiation emitted by objects, providing greater stability under low-light and challenging illumination conditions, but it is also constrained by sensitivity to environmental factors, limited spatial resolution, and insufficient contour representation. Consequently, in practical applications, thermal imaging is better suited as a complementary modality that supplies region-level thermal cues rather than fine-grained geometric details, thereby alleviating the performance degradation of visible-light imaging in complex scenarios. By detecting the thermal radiation emitted by objects, TIR sensors can robustly capture object shapes and contours under low light, backlight, occlusion, and harsh environmental conditions. As illustrated in Figure 1, thermal imaging complements visible-spectrum images by offering an additional perceptual dimension, thereby enhancing model robustness and generalization in complex environments. Consequently, multimodal fusion techniques have become a research focus in recent years. By integrating RGB and TIR modalities, these methods substantially enhance semantic segmentation [9,10], object detection [11,12], and scene understanding [13], particularly in real-world applications such as nighttime autonomous driving [14,15], occlusion-aware surveillance [16], and disaster response [17], where thermal cues provide critical perceptual information unavailable in the visible spectrum.

Most existing multimodal fusion networks adopt a bilateral architecture to perform hierarchical and staged semantic integration of TIR and RGB information. Independent encoding branches are designed to extract modality-specific features, which are then fused via feature alignment, attention mechanisms, or cross-modality complementary modules. These dual-modality networks generally fall into two categories: direct stacking fusion and specialized structural fusion. The former achieves pixel-level fusion through vector alignment in a stage-wise or cross-stage manner, while the latter employs dedicated cross-modal interaction modules or guided decoders to enable fine-grained feature complementation while preserving modality-specific characteristics.

However, existing RGB-T segmentation networks [18,19,20,21,22,23] still face notable limitations. Current fusion strategies fail to adequately model the local entropy variations and global discontinuities inherent in thermal imagery, where intensity fluctuations and structural fragmentation arise from material properties, temperature differences, and environmental interference. As illustrated in Figure 1, unstable thermal responses (e.g., pedestrian occlusion by clothing or uneven vehicle heat distribution) introduce modality-specific noise, misleading attention mechanisms, and degrading segmentation accuracy and boundary quality. Moreover, most methods adopt rigid stage-wise fusion, neglecting dynamic modality contributions and multi-scale integration, which ultimately restricts the decoder’s capability for precise mask reconstruction.

To address these challenges, we propose Wavelet-CNet, a bilateral segmentation network that integrates wavelet-based cross-modal fusion with cross-scale detail enhancement. Different from recent RGB-T fusion architectures that treat frequency information implicitly or adopt uniform multi-scale aggregation, Wavelet-CNet explicitly decomposes thermal features in the wavelet domain and introduces modality-guided cross-scale enhancement, enabling frequency-aware structural guidance and dynamic scale interaction to address thermal entropy variation and structural discontinuity in a principled manner. The framework incorporates two key components: the Wavelet Cross Fusion Module (WCFM) and the Cross-Scale Detail Enhancement Module (CSDEM). WCFM decomposes TIR features into orthogonal wavelet components, extracting high-frequency edge details while preserving low-frequency semantic structures, which are then aligned and interacted with RGB features to enhance fine-grained guidance. Meanwhile, CSDEM injects cross-scale TIR features into the encoding pathway, employing spatial–channel recalibration and semantic concentration to dynamically aggregate multi-scale context and reinforce structural details. Together, WCFM and CSDEM mitigate thermal-induced feature misalignment while preserving modality-specific cues, enabling structurally coherent and semantically consistent RGB-T representations that improve segmentation accuracy and boundary quality.

Our main contributions are summarized as follows:

(1) To better leverage TIR information for guiding and integrating with the RGB modality, we propose Wavelet-CNet, an RGB-T segmentation network based on a bilateral encoder architecture, and validate its effectiveness on the public MFNet and PST900 datasets.

(2) We introduce the WCFM to extract multi-granularity self-attention from dual-modal inputs and achieve efficient fusion. WCFM reconstructs RGB and TIR modality features in the wavelet domain and exploits the local differences between low- and high-frequency components to generate wavelet cross-attention. A Global-FFN is further employed to reduce global redundancy via channel attention.

(3) The CSDEM performs cross-modulation between cross-scale TIR features and features from preceding stages, embedding them into the feedforward encoding process to form spatial and channel-level detail reconstruction pathways with semantic concentration. This guides the RGB branch’s semantic refinement, enhancing local structural detail and completing global semantic representations.

The remainder of this paper is organized as follows: Section 2 reviews related work on RGB and RGB-T semantic segmentation. Section 3 presents the proposed Wavelet-CNet framework in detail. Section 4 reports and analyzes comparative and ablation experiments. Finally, Section 5 concludes the paper.

## 2. Related Work

### 2.1. RGB Semantic Segmentation

Long et al. [24] pioneered end-to-end semantic segmentation with Fully Convolutional Networks (FCNs), replacing patch-wise classification with dense prediction. Subsequent works extended the encoder–decoder paradigm: U-Net [25] introduced symmetric skip connections for fine-grained segmentation with limited data; PSPNet [26] employed pyramid pooling to capture multi-scale global context; and RefineNet [27] fused shallow spatial cues with high-level semantics via chained residual pooling to refine predictions.

With the adoption of attention mechanisms, numerous segmentation models have further improved feature representation. PSANet [28] modeled global spatial dependencies through adaptive point-wise attention, while DANet [29] captured channel-wise and spatial semantic relations using dual self-attention. OCNet [30] aggregated object-level context by computing pixel similarities, and SANet [31] enhanced complex scene understanding via spatial attention and multi-scale fusion. PIDNet [32] incorporated boundary attention to coordinate detail and context branches, alleviating feature overshoot during fusion.

Recent research has increasingly shifted from convolutional architectures toward transformer-based designs, leveraging their strong global modeling capability for semantic segmentation. Early works introduced transformers as encoders with CNN decoders for spatial recovery [33], while Segmenter [34] established a fully transformer-based encoder–decoder framework. SegFormer [35] adopted a hierarchical, position-free transformer with lightweight MLP decoding to reduce computational cost, and RTFormer [36] achieved real-time performance via GPU-efficient attention. TransUNet [37] further combined CNNs with transformers in a U-shaped architecture for enhanced contextual modeling.

Despite their success on natural images, these single-modal methods degrade markedly under adverse conditions such as low illumination, weather disturbances, and occlusions, where visible-spectrum ambiguity hampers semantic discrimination. Consequently, incorporating auxiliary modalities is essential to alleviate semantic bias and improve robustness in challenging environments.

### 2.2. RGB-T Semantic Segmentation

Although single-modal segmentation methods based on RGB images can address most real-world detection and quantification tasks, the inherent visual limitations of visible light imaging prevent accurate acquisition of target objects under challenging environmental conditions. Therefore, compensating RGB images with contour and semantic information captured by thermal infrared sensors is particularly crucial. Ha et al. [18] pioneered the use of a bilateral architecture combined with lightweight mini-inception modules to achieve real-time multispectral segmentation for autonomous driving and established a pixel-level annotated urban scene RGB-T dataset. RTFNet [19] performed progressive pixel-level fusion of thermal features into the RGB encoding stages and restored feature resolution through five decoding stages. Sun et al. [20] proposed a hierarchical integration of dual-modal features and introduced Monte Carlo dropout techniques to construct a Bayesian uncertainty estimation algorithm.

The aforementioned methods adopted element-wise summation for cross-modal feature fusion within bilateral structures. To better address the feature discrepancies between RGB and thermal branches, several works have designed dedicated fusion modules. Zhang et al. [21] introduced a bidirectional image translation network to mitigate modality discrepancies and performed adaptive channel-weighted multimodal feature selection. Deng et al. [38] developed stage-wise feature-enhanced attention modules to mine and enhance multilevel features from both channel and spatial perspectives, emphasizing spatial information and attention in high-resolution RGB-T features. Liang et al. [22] proposed a TIR-led detail enhancement module and an RGB-led semantic enhancement module to independently strengthen shallow detail and deep semantic features, respectively, and constructed a multi-supervised three-branch decoder to refine spatial information. Li et al. [23] employed saliency detection to generate pseudo-labels supervising feature learning and utilized hierarchical feature fusion strategies to integrate multilevel representations. Yi et al. [39] proposed an attention-guided RGB-T segmentation framework with staged fusion and consistency learning for dual-spectrum UAV-based hot spring fluid segmentation, demonstrating the effectiveness of multimodal fusion in handling irregular boundaries and thermal uncertainty.

Despite notable advancements, prior research predominantly relies on conventional CNN frameworks and specialized attention mechanisms, which are primarily tailored to extract textures, colors, and global correlations from natural RGB images. These methods largely neglect the intrinsic local information entropy present in thermal images. Consequently, the fusion of complementary information is often disrupted by discontinuous local temperature variations, impeding the comprehensive extraction of discriminative features from critical thermal regions and ultimately limiting the segmentation performance of multimodal fusion networks.

## 3. Method

**Figure 2:**
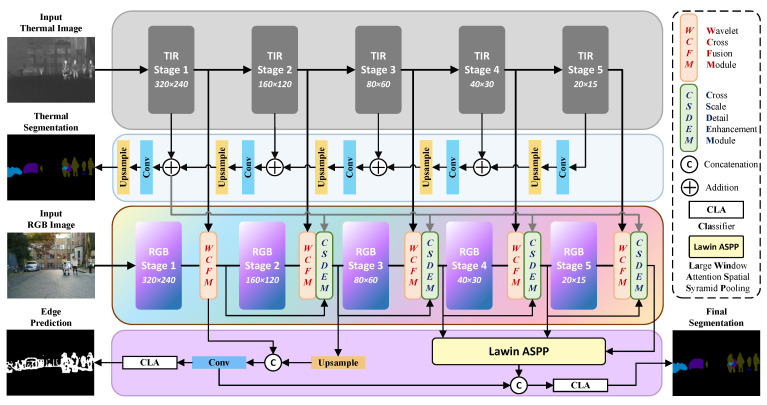
The overall architecture of Wavelet-CNet. The encoding phase of Wavelet-CNet consists of RGB and TIR encoder branches, both built upon the pre-trained ResNet50. The RGB encoder incorporates the Wavelet Cross Fusion Module (WCFM) and the Cross-Scale Detail Enhancement Module (CSDEM), which respectively fuse attention and detailed information from the TIR stage. Additionally, a TIR stage-wise upsampling decoder is constructed for semantic correction of thermal features. The segmentation decoder at the final stage includes an edge-aware head and a multi-scale semantic integration head, corresponding to the edge detection output and the final segmentation prediction, respectively.

The proposed Wavelet-CNet comprises two encoder branches and a decoder branch, including an RGB encoder, a TIR encoder, a TIR stage-wise upsampling decoder, and an Edge-Aware Lawin ASPP Decoder. The Wavelet Cross Fusion Module facilitates complementary information fusion between corresponding stages of the RGB and TIR encoders, while the Cross-Scale Detail Enhancement Module introduces global contextual information into each stage to enhance detail representation. The overall network architecture and workflow are illustrated in Figure 2.

### 3.1. Architecture Overview

As shown in Figure 2, Wavelet-CNet adopts a bilateral ResNet50-based architecture with two branches for thermal and RGB encoding. Given inputs $I^{TIR}\in R^{1\times H\times W}$ and $I^{RGB}\in R^{3\times H\times W}$, both branches pass through S(S=1,2,3,4,5) residual stages, producing feature maps $F_{S}^{TIR}$ and $F_{S}^{RGB}$ of identical dimensions, where $C_{S}$ denotes the channel number at each stage. The RGB branch is treated as the primary encoder since most spatial correspondences are more reliably captured in RGB.

Conventional RGB-T fusion strategies often operate purely in spatial or channel domains, which tends to blur high-frequency structures, introduce cross-modal misalignment, and limit fine-grained correlation modeling under thermal entropy variation. To address these issues, thermal features are integrated into the RGB stream via the Wavelet Cross Fusion Module (WCFM) and Cross-Scale Detail Enhancement Module (CSDEM). WCFM explicitly decomposes TIR features into frequency components, enabling structure-aware thermal guidance, while CSDEM injects cross-scale TIR cues to dynamically enhance multi-scale details. Together, they mitigate thermal-induced discontinuity and improve structural consistency during multimodal fusion.

During the decoding phase, we have designed a TIR decoder that fully corresponds to the TIR encoder and integrates semantic information with the encoded feature outputs through a U-shaped architecture. The TIR decoder generates the thermal segmentation result $S^{TIR}$, which is used to capture and correct pixel-level positional inaccuracies introduced by the pure thermal source information. At the lowest level of Wavelet-CNet, we adopt the edge-aware Lawin ASPP [40] decoder from SGFNet [41], which simultaneously constructs a binary edge detection head supervised by edge annotations and a segmentation head incorporating multi-scale high-level semantics, yielding the category-independent edge map $S^{edge}$ and the final segmentation output $S^{final}$, respectively.

### 3.2. Wavelet Cross Fusion Module

**Figure 3:**
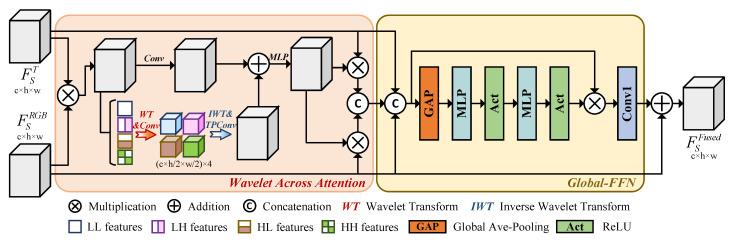
The framework of the Wavelet Cross Fusion Module (WCFM). The WCFM consists of Wavelet Across Attention and Global-FFN. Wavelet Across Attention integrates RGB and thermal infrared information, extracting attention mechanisms, while Global-FFN merges features for reconstruction.

The Wavelet Cross Fusion Module is responsible for merging and reorganizing the dual-path RGB-T features, outputting the fused features. As shown in Figure 3, WCFM consists of Wavelet Across Attention and Global-FFN. Wavelet Across Attention performs channel-wise fusion of the bimodal features and reconstructs the attention feature map in the wavelet domain.

Specifically, given the bimodal features $F_{S}^{TIR},F_{S}^{RGB}\in R^{c\times h\times w}$ of the same dimension at the same stage, we first perform feature fusion:

(1) $$F_{WT_{\_}in}=F_{S}^{TIR}⊗F_{S}^{RGB}$$

Here, ⊗ represents the Hadamard product, with $F_{WT_{\_}in}$ and $\left(F_{S}^{TIR},F_{S}^{RGB}\right)$ maintaining consistent dimensions. Subsequently, we apply an efficient and simple first-order Haar wavelet transform to decompose it into one low-frequency signal and three high-frequency signals. In the two-dimensional feature space, we construct the following four sets of filters and perform convolution with a stride of 2:

(2) $$\begin{array}{c} F_{LL}=\frac{1}{2}\left[\begin{array}{cc} 1 & 1\\ 1 & 1 \end{array}\right],F_{LH}=\frac{1}{2}\left[\begin{array}{cc} 1 & -1\\ 1 & -1 \end{array}\right],\\ F_{HL}=\frac{1}{2}\left[\begin{array}{cc} 1 & 1\\ -1 & -1 \end{array}\right],F_{HH}=\frac{1}{2}\left[\begin{array}{cc} 1 & -1\\ -1 & 1 \end{array}\right] \end{array}$$

Here, $F_{LL}$ represents the low-pass filter, and $F_{LH}$, $F_{HL}$, and $F_{HH}$ correspond to the high-pass filters in the horizontal, vertical, and diagonal directions, respectively. The convolution outputs corresponding to the four distinct directional filters are as follows:

(3) $$\begin{array}{cc} f_{WT}\left(F_{WT_{\_}in}\right)=\left[F_{LL},F_{LH},F_{HL},F_{HH}\right]\\ =\gamma_{Conv}([F_{LL},F_{LH},F_{HL} & ,F_{HH}],F_{WT_{\_}in}) \end{array}$$

Here, $\gamma_{conv}$ represents a convolution with a stride of 2 and kernel $\left[F_{LL},F_{LH},F_{HL},F_{HH}\right]$. The wavelet transform decomposition based on this kernel is orthogonal, maintaining the dimensions as c×h/2×w/2. Subsequently, the components from the four directions are subjected to an inverse wavelet transform to restore the original feature dimensions:

(4) $$\begin{array}{c} f_{IWT}\left(f_{WT}\left(F_{WT_{\_}in}\right)\right)=\gamma_{TPConv}(\left[F_{LL},F_{LH},F_{HL},F_{HH}\right],\\ \left[F_{LL},F_{LH},F_{HL},F_{HH}]\right) \end{array}$$

Here, $\gamma_{TPConv}$ denotes the transposed convolution with a stride of 2 and kernel $[F_{LL}$, $F_{LH}$, $F_{HL}$, $F_{HH}]$, employed for feature upsampling. The convolution and transposed convolution operations in both $f_{WT}$ and $f_{IWT}$ in the wavelet domain result in a doubled receptive field, while executing the first-order wavelet transform only slightly increases the parameter count, thus achieving exponential growth in receptive field with minimal parameters. Moreover, this process better captures low-frequency information compared to standard convolution, as the directional orthogonal decomposition compels the convolution to learn this component and its corresponding recovery in the transposed convolution. The high-frequency components in the remaining three directions are similarly enhanced relative to standard convolution. Additionally, a standard convolution layer is employed as a residual connection, and the merged features undergo attention reconstruction via an MLP operation. The wavelet transform attention features we propose can be expressed as follows:

(5) $$\begin{array}{c} F_{WTatt_{\_}map}=\gamma_{MLP}(f_{IWT}\left(f_{WT}\left(F_{WT_{\_}in}\right)\right)\\ +\gamma_{Conv}^{5\times 5}\left(F_{WT_{\_}in}\right)) \end{array}$$

Here, $\gamma_{Conv}^{5\times 5}$ represents a convolution with a kernel size of 5, and $\gamma_{MLP}$ denotes a convolution with a kernel size of 1, used to replace linear operations. The entire process of the Wavelet Cross Fusion Module can be defined as follows:

(6) $$\begin{array}{c} \Gamma_{WCSM}\left(F_{S}^{TIR},F_{S}^{RGB}\right)=C[\left(F_{WTatt_{\_}map}⊗F_{S}^{TIR}\right),\\ \left(F_{WTatt_{\_}map}⊗F_{S}^{RGB})\right] \end{array}$$

Here, C represents the concatenation of features along the channel dimension. The Wavelet Cross Fusion Module effectively extends the application of convolutional networks to thermal modality images by integrating directional high-frequency domain spatial features, thereby capturing the shape of the main object corresponding to concentrated heat, rather than merely surface texture features. Simultaneously, the wavelet-domain attention derived from low-frequency spatial components serves to attenuate noise, corrosion, interference, and cluttered backgrounds. The four directional wavelet components enhance the perceptual field and increase semantic robustness, concurrently facilitating the reconstruction of both RGB and thermal infrared features.

On the other hand, the output of WCSM is concatenated only along the channel dimension. This process results in channel-redundant $F_{WT_{\_}out}\in R^{2c\times h\times w}$ where simple pixel-wise summation and convolutional dimensionality reduction methods may erroneously introduce noise from the dual modalities. This issue arises primarily due to the significant differences between modalities: the thermal infrared modality contains thermal source information, which appears as a cluttered background in the RGB space, while in contrast, the texture-rich low-temperature objects in the RGB space blend with the background in thermal infrared images. To address this, we construct a Global-FFN that reduces global information redundancy while performing high-level feature reconstruction and channel reduction on $F_{WT_{\_}out}$. This structure is illustrated in Figure 3, where we first obtain the concatenated features:

(7) $$F_{Concat}=C\left[F_{WT_{\_}out},F_{S}^{TIR},F_{S}^{RGB}\right)]$$

We apply global adaptive average pooling to $F_{Concat}\in R^{4c\times h\times w}$ to obtain channel-wise keypoint information. This is followed by two MLP layers with interspersed activation functions for linear recombination, resulting in the attention weights for each corresponding channel. These weights are then used to restore the feature input by multiplying with element-wise across channels. At the end of the Global-FFN, we incorporate a convolution with a kernel size of 1 to reduce and restore the dimensionality of the channels. The process of the Global-FFN can be expressed as follows:

(8) $$\begin{array}{c} \Gamma_{Global-FFN}\left(F_{Concat}\right)=\gamma_{Conv}^{4c\to c}(\gamma_{MLP}(\sigma (\gamma_{MLP}\\ \left(\sigma \left(GAP\left(F_{Concat}\right)\right)\right)))⊗F_{Concat}) \end{array}$$

Here, $\gamma_{conv}^{4c\to c}$ denotes a dimensionality-reduction convolutional layer with an input channel count of 4c and an output channel count of *c*. GAP denotes global average pooling. σ represents the ReLU activation function. Ultimately, we directly connect $F_{S}^{RGB}$ to the output of the WCFM to obtain $F_{S}^{\mathrm{Fused}}\in R^{c\times h\times w}$. The process of the WCFM can be formalized as follows:

(9) $$\begin{array}{c} F_{S}^{Fused}=\Gamma_{WCFM}\left(F_{S}^{TIR},F_{S}^{RGB}\right)=F_{S}^{RGB}\\ +\Gamma_{Global-FFN}(C[\Gamma_{WCSM}\left(F_{S}^{TIR},F_{S}^{RGB}\right)\\ ,F_{S}^{TIR},F_{S}^{RGB}]) \end{array}$$

### 3.3. Cross-Scale Detail Enhancement Module

The RGB pathway is limited in capturing local details due to variations in illumination, occlusion, and single-scale constraints. Although WCFM alleviates the field-of-view deficiency in the visible spectrum by integrating multi-frequency thermal semantic information, local overexposure and underexposure regions introduce lighting and shadow noise. Moreover, as feature resolution decreases progressively, the base encoder’s ability to capture fine edges and discontinuous textures is significantly diminished with the expansion of the receptive field, leading to the gradual loss of structural information in $F_{S}^{Fused}$ during downsampling. This adversely affects the model’s capacity to accurately represent blurred boundaries and complex structures. To address this issue, we propose the Cross-Scale Detail Enhancement Module, which enhances the fine-grained perception of $F_{S}^{Fused}$ by incorporating high-resolution semantic features from the TIR decoder stage with intermediate RGB features.

**Figure 4:**
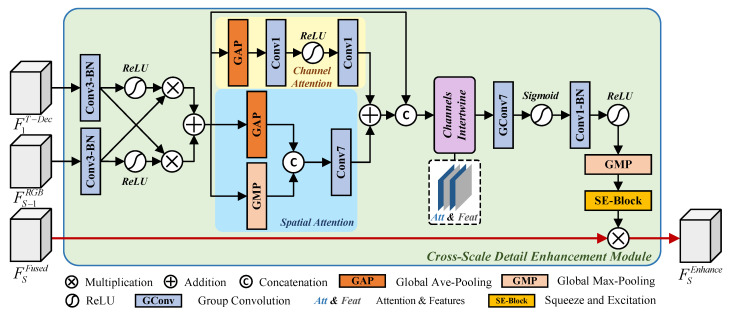
The framework of the Cross-Scale Detail Enhancement Module (CSDEM). The CSDEM consists of an upper cross-attention cluster pathway and a lower red main pathway. The cross-attention cluster pathway integrates the TIR encoder output with RGB features across scales and applies various attention mechanisms to generate refined self-attention, which is then combined with the fused features via dot product to produce the refined output.

As illustrated in Figure 4, CSDEM comprises two primary pathways: the upper cross-attention cluster pathway and the lower red enhanced feedforward pathway. We first perform cross-modal fusion between the high-resolution TIR decoder features $F_{1}^{T-Dec}$ and the preceding RGB features $F_{S-1}^{RGB}$ to obtain the attention input features $F_{S}^{Att\_in}$.

(10) $$\begin{array}{c} F_{S}^{Att\_in}=\gamma_{Conv-BN}^{3\times 3}\left(F_{1}^{T-Dec}\right)⊗\sigma \left(\gamma_{Conv-BN}^{3\times 3}\left(F_{S-1}^{RGB}\right)\right)\\ +\gamma_{Conv-BN}^{3\times 3}\left(F_{S-1}^{RGB}\right)⊗\sigma \left(\gamma_{Conv-BN}^{3\times 3}\left(F_{1}^{T-Dec}\right)\right) \end{array}$$

Here, $\gamma_{Conv-BN}^{3\times 3}$ denotes a 3×3 convolutional layer followed by batch normalization.

Subsequently, $F_{S}^{Att\_in}$ is processed through two attention branches: channel attention and spatial attention. The channel attention branch computes channel-wise vectors to recalibrate inter-channel weights, while the spatial attention branch generates local spatial weight maps. By fully integrating the attention maps from both branches, we ensure effective multi-granularity information interaction along the same semantic direction.

(11) $$\begin{array}{c} F_{S}^{Att\_fused}=\gamma_{Conv}^{1\times 1}\left(\max\left(0,\gamma_{Conv}^{1\times 1}\left(\mathrm{GAP}\left(F_{S}^{Att\_in}\right)\right)\right)\right)\\ +\gamma_{Conv}^{7\times 7}\left(C\left[\mathrm{GAP}\left(F_{S}^{Att\_in}\right)+\mathrm{GMP}\left(F_{S}^{Att\_in}\right)\right]\right) \end{array}$$

Here, GMP indicates global max pooling.

We interleave the fused attention feature $F_{S}^{Att\_fused}$ with the input features through channel-wise alignment, ensuring that each original feature channel is positioned adjacent to its corresponding attention map, i.e., $\left[C_{1}^{Att\_in},C_{1}^{Att\_fused},\ldots ,C_{n}^{Att\_in},C_{n}^{Att\_fused}\right]$. By integrating grouped convolutions, the attention-enhanced features are leveraged to guide the spatial activation of original features within local pixel regions. This collaborative mechanism compensates for the backbone network’s limited sensitivity to weak textures and complex boundaries, thereby enhancing the representation of fine-grained texture edges and structural contours. Meanwhile, it strengthens attention-guided semantic alignment while maintaining computational efficiency.

Additionally, we reduce the channels of the detail-enhanced features and apply global average pooling followed by Squeeze and Excitation [42] operations to concentrate and refine the semantic output $F_{S}^{Att}$. This process adaptively recalibrates the global contextual information through channel suppression and activation, enabling precise localization and nonlinear dependency reconstruction of fine-grained features. Finally, we perform an element-wise multiplication between the detail-aware attention map $F_{S}^{Att}$ and the fused features $F_{S}^{Fused}$ to obtain the enhanced representation $F_{S}^{Emhance}$.

### 3.4. Fusion Loss Function

For the multi-input, multi-output Wavelet-CNet, we employ a composite loss function to guide the optimization objectives at each output stage. Among the three output targets shown in Figure 2, we first establish a weighted cross-entropy loss and Lovasz-softmax loss between the final segmentation result and the ground truth to address class pixel imbalance, thereby compelling the network to focus on more challenging-to-classify pixels. This process can be expressed as follows:

(12) $$\begin{array}{c} L_{sem}^{final}=-\frac{1}{N}\sum_{i=1}^{N}w_{y_{i}}\cdot \log\left(p_{i,y_{i}}\right)+\frac{1}{C}\sum_{c=1}^{C}{\bar{\Delta }}_{\mathrm{Jac}}\left(m^{\left(c\right)}\right) \end{array}$$

Here, *N* represents the total number of valid pixels, $y_{i}\in \left\{1,...,C\right\}$ denotes the true class of the *i*-th pixel, $p_{i,y_{i}}$ is the predicted probability for class $y_{i}$, $W_{y_{i}}$ is the weight of class $y_{i}$, and $m^{\left(c\right)}$ is the ranking error vector of the gradient of the IoU loss function for the *c*-th class.

In the final encoding stage of the network, we utilize shallow feature information for semantic multi-class output, performing edge extraction for all classes except the background and constructing a binary cross-entropy loss as the edge loss $L_{edge}$. Additionally, to enhance the segmentation performance of the TIR branch in the guided bilateral structure, we apply a weighted cross-entropy loss $L_{sem}^{TIR}$ for the thermal infrared segmentation results. These four loss functions are combined through weighted summation to form the hybrid loss function:

(13) $$L_{fused}=\alpha L_{sem}^{final}+\beta L_{sem}^{TIR}+\gamma L_{edge}$$

Based on empirical values, we set α = 1, β = 0.5, and γ = 2.

## 4. Experiments

### 4.1. Experimental Setting

(1) Datasets: We trained and evaluated both the proposed and comparative methods using the publicly available RGB-T segmentation datasets MFNet [18] and PST900 [43], assessing various performance metrics. The MFNet dataset contains pixel-level annotations for vehicle-driving videos captured in urban environments, with a total of 1569 images, of which 820 were taken during the day and 749 at night. MFNet categorizes common driving obstacles into eight classes: car, pedestrian, bicycle, curve, parking spot, guardrail, traffic cone, and pothole, with an image resolution of 480 × 640. We constructed training, validation, and test sets for MFNet, conducting separate tests for daytime and nighttime scenes. The PST900 dataset comprises paired RGB and thermal images for multi-spectral segmentation, containing 894 synchronized and calibrated image pairs with a resolution of 1280 × 720. It is semantically divided into five classes: fire extinguisher, backpack, hand drill, and survivor.

(2) Implementation Details: We implemented the proposed Wavelet-CNet within the PyTorch 1.13.1 framework and conducted all comparative and ablation experiments on an NVIDIA RTX A6000 GPU. We utilized the training and test sets provided by the datasets, applying data augmentation techniques such as random horizontal flipping, scaling, color jittering, and random cropping. The batch size was set to 6, with an initial learning rate of $5\times 10^{-5}$, a weight decay factor of $5\times 10^{-4}$, and learning rate smoothing decay. The training process was optimized using the Ranger optimizer, and a total of 220 epochs were executed.

(3) Evaluation Metrics: We evaluate the effectiveness of Wavelet-CNet by reporting pixel accuracy (Acc), mean accuracy (mAcc), intersection over union (IoU), and mean intersection over union (mIoU) in both comparative and ablation experiments.

### 4.2. Comparison with State-of-the-Art Methods

We conducted comparative experiments between Wavelet-CNet and several state-of-the-art and representative RGB-T semantic segmentation methods. During the experimental phase, we constructed identical training and testing sets based on MFNet and PST900 and standardized the default training parameters as much as possible. The segmentation results and evaluation metrics of the compared methods were obtained from their publicly available code.

**Comparison on the MFNet Dataset:** Table 1 presents the quantitative results of the proposed Wavelet-CNet in comparison with all RGB-T segmentation networks. Compared to other methods, our approach achieves the highest IoU for the Curve category. While its performance on the remaining categories is not the most accurate, the IoU scores remain relatively balanced across all classes. This demonstrates Wavelet-CNet’s ability to model features uniformly across different semantic categories, effectively avoiding overfitting to dominant classes and exhibiting strong generalization and robustness. Ultimately, our method achieves the best overall mIoU score. Meanwhile, we removed the infrared modality to comparatively evaluate the proposed RGB-T network. As observed, for thermally salient targets such as Person and detail-sensitive categories like Guardrail, Wave-CNet with infrared integration achieves more stable and substantially superior IoU performance, thereby validating the thermal modality as an effective complement for target perception and boundary modeling under challenging illumination conditions.

**Figure 5:**
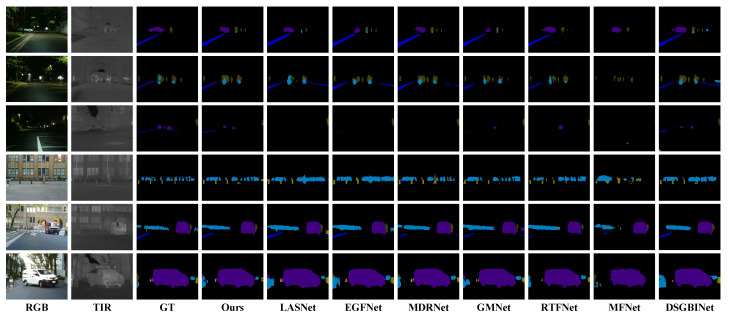
Visual segmentation results of daytime and nighttime scenes in MFNet dataset using Wavelet-CNet and other methods.

Figure 5 shows qualitative comparisons across various methods on the MFNet dataset under both daytime and nighttime conditions. Our method demonstrates accurate detection of thermal and small-scale objects in nighttime scenes—such as cyclists, vehicles, and the Curve category—achieving enhanced edge delineation and semantic completeness through the incorporation of TIR information. In daytime scenarios, our approach yields fine-grained representations, where detail enhancement enables clearer separation of complex instances such as stacked bicycles, pedestrians near vehicles, and color cones. In contrast, other methods tend to exhibit semantic adhesion and misclassification for small objects.

As shown in Table 2, the proposed method achieves the best overall performance under both daytime and nighttime conditions. It attains 74.2% Acc and 50.6% IoU during daytime, surpassing SGFNet by +3.5% Acc and +1.4% IoU, indicating improved fine-grained modeling. At night, our method achieves 70.5% Acc and 58.3% IoU, ranking second in IoU and closely approaching SGFNet (58.4%), while maintaining competitive accuracy. Compared with earlier RGB-T frameworks, nighttime IoU improves by over 14%, validating the effectiveness of thermal-aware frequency decomposition and cross-scale enhancement. Although SGFNet slightly outperforms our method in nighttime IoU, our approach demonstrates more consistent region localization and boundary preservation, confirming the robustness of Wavelet-CNet in challenging low-light environments.

As shown in Table 3, our method achieves the highest mIoU with moderate computational cost. Compared with heavier models such as RTFNet152 and CCFFNet50, our approach delivers superior accuracy with substantially fewer parameters and FLOPs. Meanwhile, it significantly outperforms lightweight models (e.g., PSTNet and MMNet), demonstrating a favorable accuracy–efficiency trade-off. These results validate the effectiveness of the proposed wavelet-based fusion and cross-scale enhancement.

**Figure 6:**
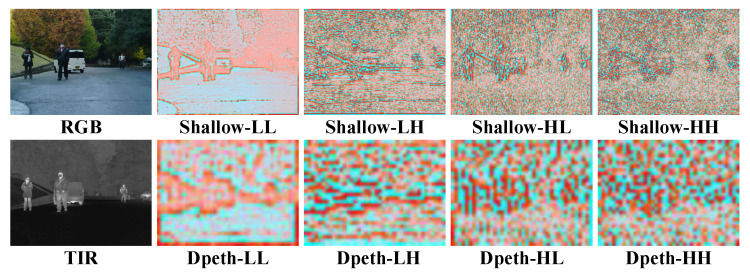
Visualization of feature maps after wavelet decomposition. The upper row illustrates the decomposition results of shallow texture features, while the lower row presents the decomposition results of deep semantic features.

Figure 6 presents the visualized wavelet-decomposed features in WCFM. In the shallow stages, the low-frequency component (LL) primarily focuses on large-scale scene elements, such as humans and vehicles, while the three high-frequency components (LH, HL, HH) effectively capture the precise boundaries of thermal objects from different directional perspectives. In deeper stages, the low-frequency features preserve the semantic focus from the forward encoding process, yet the absence of edge information renders the high-frequency components less effective. Overall, the refined frequency-domain features exhibit anisotropic characteristics across stages, highlighting the role of edge and texture cues in guiding semantic localization during encoding.

**Comparison on the PST900 Dataset:** As shown in Table 4, our method achieved the highest IoU scores in three out of the five categories in the dataset, demonstrating highly competitive performance. Notably, for the high-thermal-source category “fire extinguisher,” the IoU significantly outperformed other methods, exceeding the CCFFNet50W network by 3.18%. Furthermore, for the remaining two categories, “Hand-drill” and “Survivor,” Wavelet-CNet exhibited balanced performance across multiple semantic tasks, without any notable weaknesses. The superior mIoU score further highlights the high performance and robustness of the proposed approach.

**Figure 7:**
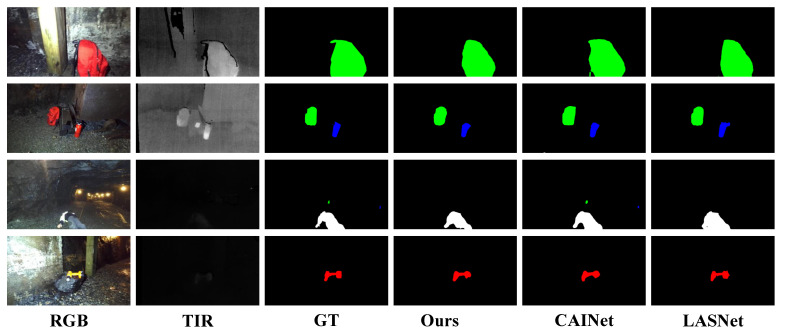
Visual segmentation results in PST900 dataset using Wavelet-CNet and other methods.

The visualization results of Wave-CNet on the PST900 dataset are shown in Figure 7. It is evident that under strong TIR signals, our method achieves the closest alignment with the ground truth, exhibiting well-defined boundaries and accurately segmented semantic regions compared to other approaches. For samples with weaker TIR signals, our method closely adheres to irregular object regions locally, benefiting from the multi-scale information fusion in CSDEM. In contrast, CAINet produces overly smooth boundaries that do not match the true object shapes, while LASNet shows a higher rate of misclassification.

**Figure 8:**
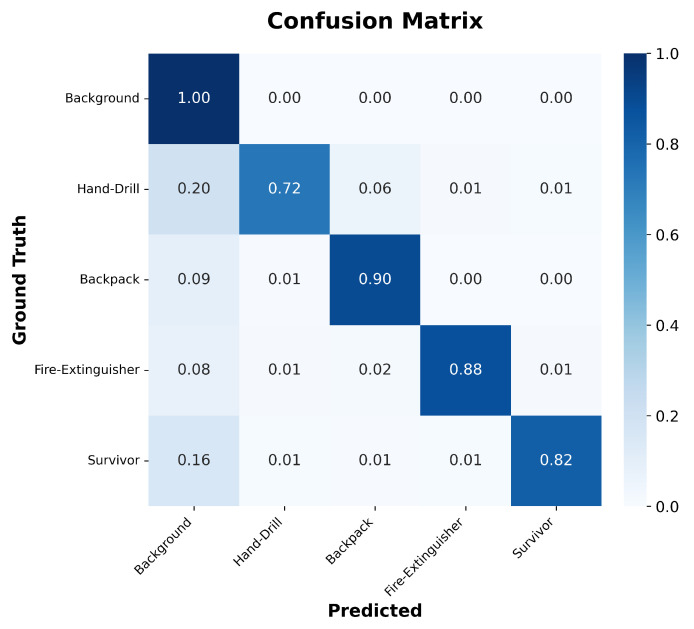
Confusion matrix of Wavelet-CNet on the PST900 test set.

To further evaluate the classification performance of the model, the confusion matrix is presented in Figure 8. As observed, the model demonstrates strong recognition capabilities for the Backpack and Fire-Extinguisher categories, achieving accuracies of 0.90 and 0.88, respectively, with minimal misclassification. However, for the Hand-Drill and Survivor categories, the classification accuracy is relatively lower, with 20% and 16% of samples misclassified as background, respectively. This suggests that local texture or shape similarities between Hand-Drill and Backpack contribute to a certain degree of confusion.

### 4.3. Ablation Studies

We conducted ablation studies on the MFNet dataset to validate the effectiveness of the proposed modules. The following configurations were evaluated: (1) baseline, (2) baseline + WCFM, (3) baseline + CSDEM, and (4) baseline + WCFM + CSDEM.

As shown in Table 5, the baseline model achieved an mIoU of only 56.1%. With the inclusion of WCFM, the mIoU improved to 57.5%, accompanied by a 2.3% increase in mAcc, indicating that WCFM effectively integrates semantic information across most categories during multimodal fusion, thereby enhancing overall segmentation performance. Adding CSDEM to the baseline raised the mIoU to 57.2%, though with a slight decline in mAcc, suggesting that CSDEM improves boundary precision for small objects with low pixel occupancy, albeit at the expense of some conflict with other categories. The final Wavelet-CNet configuration achieved the best performance with an mIoU of 58.3%.

Table 6 reports the complexity analysis of each module in the ablation study. Compared with No. 4, the complete architecture achieves a 2.2% performance gain with only an additional 0.41 M parameters and 4.15 GMac FLOPs. As indicated by the results of No. 2 and No. 3, most of the parameter increase is attributed to the construction of WCFM, which also yields a notable improvement of 1.4% in mIoU. Furthermore, on a single RTX 3090 GPU, the proposed method maintains an inference speed of 11.41 FPS, demonstrating its capability to meet real-time requirements in practical scenarios.

## 5. Conclusions

This paper proposes Wavelet-CNet, a bilateral RGB-T fusion framework that combines wavelet-domain cross-modal fusion with cross-scale detail enhancement. Unlike most existing RGB-T methods, which perform fusion mainly in the spatial domain, our approach explicitly decomposes RGB and TIR features into low-frequency semantic components and high-frequency structural details in the wavelet domain. This design enables thermal cues to more effectively guide boundary reconstruction and mitigates semantic–structural entanglement, especially under low-light or thermally dominant conditions. Moreover, the Cross-Scale Detail Enhancement Module aligns multi-scale RGB features with high-resolution TIR decoder representations, facilitating consistent information propagation and preserving fine-grained boundaries. Experiments on MFNet and PST900 demonstrate that Wavelet-CNet achieves improved accuracy and robustness, validating the effectiveness of wavelet-domain fusion and modality-guided cross-scale enhancement for RGB-T semantic segmentation.

## Figures and Tables

**Table 1:** Quantitative comparisons (%) on the test set of the MFNet dataset. The value 0.0 indicates that there are no true positives. “-” means that the authors do not provide the corresponding results. The top three results in each column are highlighted in red, blue, and green.

Methods	Car		Person		Bike		Curve		Car Stop		Guardrail		Color Cone		Bump		mAcc	mIoU
	Acc	IoU	Acc	IoU	Acc	IoU	Acc	IoU	Acc	IoU	Acc	IoU	Acc	IoU	Acc	IoU		
MFNet	77.2	65.9	67.0	58.9	53.9	42.9	36.2	29.9	19.1	9.9	0.1	8.5	30.3	25.2	30.0	27.7	45.1	39.7
RTFNet50	91.3	86.3	78.2	67.8	71.5	58.2	69.8	43.7	32.1	24.3	13.4	3.6	40.4	26.0	73.5	**57.2**	62.2	51.7
RTFNet152	93.0	87.4	79.3	70.3	76.8	62.7	60.7	45.3	38.5	29.8	0.0	0.0	45.5	29.1	74.4	**55.7**	63.1	53.2
PSTNet	-	76.8	-	52.6	-	55.3	-	29.6	-	25.1	-	**15.1**	-	39.4	-	45.0	-	48.4
MMNet	-	83.9	-	69.3	-	59.0	-	43.2	-	24.7	-	4.6	-	42.2	-	50.7	62.7	52.8
MLFNet	-	82.3	-	68.1	-	**67.3**	-	27.3	-	30.4	-	**15.7**	-	**55.6**	-	40.1	-	53.8
FuseSeg	93.1	**87.9**	81.4	71.7	**78.5**	**64.6**	68.4	44.8	29.1	22.7	63.7	6.4	55.8	46.9	66.4	47.9	70.6	54.5
ABMDRNet	94.3	84.8	90.0	69.6	75.7	60.3	64.0	45.1	44.1	33.1	31.0	5.1	61.7	47.4	66.2	50.0	69.5	54.8
FEANet	93.3	**87.8**	82.7	71.1	76.7	61.1	65.5	**46.5**	26.6	22.1	**70.8**	6.6	**66.6**	**55.3**	**77.3**	48.9	73.2	55.3
EGFNet	**95.8**	87.6	89.0	69.8	**80.6**	58.8	**71.5**	42.8	48.7	33.8	33.6	7.0	**65.3**	48.3	71.1	47.1	72.7	54.8
LASNet	94.9	84.2	81.7	67.1	**82.1**	56.9	70.7	41.1	**56.8**	**39.6**	59.5	**18.9**	58.1	48.8	**77.2**	40.1	**75.4**	54.9
GCNet	94.2	86.0	89.6	**72.0**	77.5	60.0	68.9	42.8	38.3	30.7	45.8	6.2	59.6	49.5	**82.1**	52.6	72.7	55.3
MDRNet	**95.2**	87.1	**92.5**	69.8	76.2	60.9	**72.0**	**47.8**	42.3	34.2	**66.8**	8.2	**64.8**	50.2	63.5	**55.0**	**74.7**	56.8
GMNet	94.1	86.5	83.0	**73.1**	76.4	61.7	59.7	44.0	**55.0**	**42.3**	**71.2**	14.5	54.7	48.7	73.1	47.4	**74.1**	**57.3**
SGFNet	94.3	**88.4**	**90.3**	**77.6**	77.9	**64.3**	67.7	45.8	37.7	31.0	57.3	6.0	64.2	**57.1**	73.4	**55.0**	73.6	**57.6**
**Ours (RGB)**	94.0	86.8	85.5	69.4	81.3	62.3	65.8	42.5	43.0	32.4	59.2	5.2	51.9	60.5	48.7	78.0	73.9	55.2
**Ours (RGB-T)**	94.2	**87.8**	**91.0**	71.9	75.6	62.8	**71.1**	**48.2**	**51.3**	**41.8**	43.2	10.1	60.7	53.7	72.8	50.7	73.2	**58.3**

**Table 2:** Quantitative comparison (%) on the MFNet test set under daytime and nighttime conditions.

Methods	Daytime		Nighttime	
	Acc	IoU	Acc	IoU
MFNet	42.6	36.1	48.9	43.9
RTFNet50	57.3	44.4	59.4	52.0
RTFNet152	60.0	45.8	60.7	54.8
MLFNet	–	45.6	–	54.9
FuseSeg	62.1	47.8	67.3	54.6
ABMDRNet	58.4	46.7	68.3	55.5
SGFNet	70.7	49.2	72.2	58.4
Ours	74.2	50.6	70.5	58.3

**Table 3:** Model complexity and inference speed were evaluated on 480 × 640 RGB-T image pairs using an RTX 3090 GPU in the comparative experiments.

Methods	Input Size	Parameter (M)	Flops (GMac)	mIoU
RTFNet50	640×480	185.24	245.71	51.7
RTFNet152	640×480	254.51	337.04	53.2
PSTNet	640×480	20.38	129.37	48.4
MMNet	640×480	23.90	95.00	52.8
ABMDRNet	640×480	64.60	194.33	54.8
CCFFNet50	640×480	71.07	345.40	57.4
Ours	640×480	125.66	147.88	58.3

**Table 4:** Quantitative comparisons (%) on the test set of PST900 dataset. The value 0.0 indicates that there are no true positives. “-” means that the authors do not provide the corresponding results. The top three results in each column are highlighted in red, blue, and green.

Methods	Background		Hand-Drill		Backpack		Fire-Extinguisher		Survivor		mAcc	mIoU
	Acc	IoU	Acc	IoU	Acc	IoU	Acc	IoU	Acc	IoU		
MFNet	-	98.63	-	41.13	-	64.27	-	60.35	-	20.70	-	57.02
PSTNet	-	98.85	-	53.60	-	69.20	-	70.12	-	50.03	-	68.36
MDRNET	99.30	99.07	90.17	63.04	**93.53**	76.27	86.63	63.47	**85.56**	71.26	91.04	74.62
EGFNet	99.48	99.26	**97.99**	64.67	**94.17**	83.05	**95.17**	71.29	83.30	74.30	**94.02**	78.51
MTANet	-	99.33	-	62.05	-	**87.50**	-	64.95	-	**79.14**	-	78.60
MFFENet	-	99.40	-	72.50	-	81.02	-	66.38	-	75.60	-	78.98
GMNet	**99.81**	99.44	90.29	**85.17**	89.01	83.82	88.28	73.79	80.86	78.36	89.61	84.12
CCFFNet50	**99.9**	99.4	89.7	**82.8**	77.5	75.8	87.6	**79.9**	79.7	72.7	86.9	82.1
DSGBINet	99.73	99.39	**94.53**	74.99	88.65	85.11	**94.78**	**79.31**	81.37	75.56	**91.81**	82.87
FDCNet	99.72	99.15	82.52	70.36	77.45	72.17	91.77	71.52	78.36	72.36	85.96	77.11
LASNet	99.77	**99.46**	91.81	**82.80**	90.80	86.48	**92.36**	77.75	83.43	75.49	91.63	84.40
CAINet	99.66	**99.50**	**95.87**	**80.30**	**96.09**	**88.02**	88.38	77.21	**91.35**	**78.69**	**94.27**	**84.74**
**Ours**	**99.86**	**99.54**	89.80	79.76	90.59	**88.03**	92.23	**83.08**	81.62	**78.43**	90.82	**85.77**

**Table 5:** Quantitative results (%) for evaluating the effectiveness of WCFM and CSDEM in Wavelet-CNet.

No.	Baseline	WCFM	CSDEM	mAcc	mIoU
1	✓	-	-	72.7	56.1
2	✓	✓	-	75.0	57.5
3	✓	-	✓	71.2	57.2
4	✓	✓	✓	73.2	58.3

**Table 6:** Model complexity and inference speed were evaluated on 480 × 640 RGB-T image pairs using an RTX 3090 GPU in the ablation study.

No.	Input Size	Parameter (M)	Flops (GMac)	FPS	mIoU
1	640×480	125.25	143.73	12.89	56.1
2	640×480	125.62	146.82	12.60	57.5
3	640×480	125.29	144.83	12.06	57.2
4	640×480	125.66	147.88	11.41	58.3

## Data Availability

The MFNet dataset can be found at https://www.mi.t.u-tokyo.ac.jp/static/projects/mil_multispectral/, accessed on 3 February 2026; the PST900 dataset can be found at https://github.com/ShreyasSkandanS/pst900_thermal_rgb, accessed on 3 February 2026.
